# Activator protein 1 (AP-1) contributes to EpCAM-dependent breast cancer invasion

**DOI:** 10.1186/bcr3070

**Published:** 2011-12-01

**Authors:** Narendra V Sankpal, John D Mayfield, Mike W Willman, Timothy P Fleming, William E Gillanders

**Affiliations:** 1 Department of Surgery, Washington University School of Medicine, Campus Box 8109, 660 South Euclid Avenue, St. Louis, Missouri, 63110, USA; 2 The Alvin J. Siteman Cancer Center at Barnes-Jewish Hospital and Washington University School of Medicine, 660 South Euclid Avenue, St. Louis, Missouri, 63110, USA

## Abstract

**Introduction:**

EpCAM is a cell-surface glycoprotein that is overexpressed in the majority of epithelial carcinomas. However, the functional role of EpCAM in regulating cancer invasion remains controversial, and the mechanism(s) underlying EpCAM-mediated regulation of breast cancer invasion remain to be defined.

**Methods:**

EpCAM expression was manipulated in breast cancer cell lines using RNA interference and cDNA expression constructs. Recombinant EpCAM was used to rescue EpCAM signaling following specific ablation of EpCAM. Protein and gene expression, invasion, transcription factor activity, and protein phosphorylation were measured using standard molecular biology techniques.

**Results:**

In loss-of-function, and gain-of-function experiments we demonstrate that EpCAM expression is associated with increased breast cancer invasion *in vitro *and *in vivo*. We demonstrate further that specific ablation of EpCAM expression is associated with decreased activator protein-1 (AP-1) transcription factor activity. Phosphoprotein analyses confirm that specific ablation of EpCAM is associated with decreased phosphorylation of the AP-1 subunit c-Jun. Recombinant soluble extracellular EpCAM (rEpCAM) is able to rescue invasion, AP-1 transcription factor activity, and c-Jun phosphorylation in a dose-dependent fashion. Pharmacologic inhibitors, and constitutively active constructs of the c-Jun N-terminal kinase (JNK) signal transduction pathway, suggest that the impact of EpCAM expression on AP-1 transcription factor activity is mediated through the JNK pathway. In functional rescue experiments, forced expression of c-Jun rescues invasion in breast cancer cells following specific ablation of EpCAM.

**Conclusions:**

These data demonstrate for the first time that EpCAM expression can influence the JNK/AP-1 signal transduction pathway, and suggest that modulation of AP-1 transcription factor activity contributes to EpCAM-dependent breast cancer invasion. These data have important implications for the design and application of molecular therapies targeting EpCAM.

## Introduction

The epithelial cell adhesion molecule (EpCAM) is a type I transmembrane protein that is localized to the basolateral membrane in the majority of normal epithelial tissues [1]. The functional role of EpCAM in cell adhesion was the focus of early studies, and EpCAM has been demonstrated to be a calcium-independent homophilic cell adhesion molecule [2]. Recent studies have also demonstrated a role for EpCAM in cell signaling, proliferation and invasion [3-7]. EpCAM is perhaps best known for the fact that it is overexpressed in the majority of human epithelial cancers including colorectal, breast, gastric, prostate, ovarian, and lung cancers [8,9]. EpCAM was the first human tumor-associated antigen to be identified with monoclonal antibodies [10], and was the first target of monoclonal antibody therapy in humans [11]. Although initial results have been disappointing, a number of second-generation molecular therapies are currently under development [12-17]. Despite this intense interest in EpCAM as a target for molecular therapy, there have been limited attempts to define the functional role of EpCAM in cancer biology.

EpCAM expression in primary cancer specimens has been studied extensively, and a number of studies in the surgical pathology literature have evaluated the association between EpCAM expression and prognosis. One inconsistency in the literature is that EpCAM expression in primary cancer specimens appears to be associated with a favorable prognosis in some cancer types, and an unfavorable prognosis in other cancer types. For instance, EpCAM expression in primary breast cancers appears to be associated with decreased patient survival [8,18-20]. However, EpCAM expression in colorectal cancer appears to be associated with improved patient survival [21]. Additional studies in other cancer types have suggested an association with improved patient survival in esophageal cancer [22], gastric cancer [23], and renal cell carcinoma [24,25], and an association with decreased patient survival in ovarian cancer [26], gall bladder cancer [27], and pancreatic cancer [28]. Although these studies are far from definitive, taken together, they do suggest a cancer type-specific role for EpCAM in cancer biology and invasion. This inconsistency is paralleled in functional studies of EpCAM biology performed *in vitro*. Loss-of-function analyses using RNA interference suggest that EpCAM expression is associated with increased invasion in breast cancer [4], and gain-of-function analyses in colorectal and lung cancers suggest that EpCAM expression is associated with decreased cancer invasion in these cancer types [29,30]. A better understanding of the relation between EpCAM and cancer invasion will clearly facilitate the rational design, and successful application of molecular therapies targeting EpCAM in epithelial carcinomas.

In this study we confirm that EpCAM expression is associated with increased breast cancer invasion *in vitro *and *in vivo*. In mechanistic studies, we demonstrate for the first time that EpCAM expression can modulate the c-Jun N-terminal kinase (JNK)/activator protein 1 (AP-1) signal transduction pathway and target genes. These observations provide important insights into the downstream mediators of EpCAM signaling, and the impact of EpCAM expression on breast cancer invasion and prognosis.

## Materials and methods

### Cell culture

The MDA-231 and MCF-7 breast cancer cell lines were obtained from the American Type Culture Collection (ATCC, Rockville, MD, USA). The CA1a breast cancer cell line was obtained from Dr. Fred Miller at Wayne State University (Detroit, MI, USA).

### RNA interference and lentivirus generation

The lentiviral construct pSicoR and related expression vectors were obtained from Dr. Tyler Jacks (Massachusetts Institute of Technology, Boston, MA, USA) [31]. Two shRNA target sequences specific for EpCAM (sh1, sh2) and a scrambled control sequence were cloned into the pSicoR-puromycin vector as previously described [32]. shRNA constructs were transfected into HEK293T cells with VSVG and Δ8.9, and viral supernatants were collected at 48 and 72 hours to transduce cells.

### Plasmids and transfection

EpCAM cDNA was cloned from the MCF-7 breast cancer cell line. The c-Jun expression construct was obtained from Dr. R. Lee at the University of Virginia (Charlottesville, VA, USA). HA-MEKK1 was obtained from Dr. J. Avruch at Massachusetts General Hospital (Cambridge, MA, USA). Constructs from the JNK signal transduction pathway including pcDNA3 FLAG-MKK7/JNK1, pcDNA3 FLAG-MKK7, and pcDNA3 FLAG-JNK1 were obtained from Addgene (Cambridge, MA, USA). Cells were transfected with FuGENE-HD (Roche, Indianapolis, IN, USA) or Lipofectamine LTX (Invitrogen, Carlsbad, CA, USA) as recommended by the manufacturer.

### Invasion assay

For invasion assays, stably transduced cells (4 × 10^4^) were added to matrigel transwell invasion chambers or control transwell chambers (BD Biosciences San Jose CA, USA) and incubated for 24 to 72 hours with chemoattractant media (MEGM Clonetics, Walkersville, MD, USA) supplemented with growth factors. Cells invading through the matrigel or control membranes were fixed using 70% ethanol, stained with 0.1% crystal violet and photographed in four fields to cover the entire area. Cells were counted from all fields by a scientist blinded to the experimental conditions.

### Tumor challenge

Six- to eight-week-old female nude mice were used for tumor challenge experiments. All mice were housed in pathogen-free facilities accredited by the Association for Assessment and Accreditation of Laboratory Animal Care. Animal use protocols were approved by the Washington University Animal Studies Committee. MDA-231 breast cancer cells stably transduced with scrambled control shRNA construct (SCR) and sh2 lentiviral shRNA constructs were harvested at 70 to 90% confluence. A 1 × 10^7 ^sample of MDA-231 cells were resuspended in matrigel and injected into nude mice in a total volume of 200 μL (*n *= 10 per group). The right flank was injected with MDA-231(SCR) cells and the left flank was injected with MDA-231(sh2) cells. Tumor size was measured by electronic calipers every three to five days until 45 days. At the time of sacrifice, tumors were stored in formalin and submitted to an institutional core facility for H&E staining and evaluation.

### Cell cycle analysis and MTT proliferation assay

Stably transduced MDA-231 cells were plated in culture media at 0.5 × 10^6 ^cells/mL. After 24 hours, the culture media was changed, and cells were pulse-labeled with BrdU (final concentration, 1 μM) for 30 minutes. Cells were washed with 1 × PBS and fixed with 70% cold ethanol. For staining, cells were washed 1 × with PBS with 0.1% BSA, and 0.1% Tween 20 and digested with DNAse for 30 minutes. The cells were washed again and then incubated with BrdU primary antibody (Becton Dickinson Immunocytometry Systems, San Jose, CA, USA) and fluorescein isothiocyanate (FITC) GAM secondary antibody (Becton Dickinson, San Jose, CA, USA). Cell cycle analyses were performed using the ModFit LT software (Topsham, ME, USA). For MTT assays, 5,000 cells were plated in 96-well plates in triplicate. After culture for the indicated time, MTT assays were preformed using a Vibrant MTT cell proliferation assay kit (Invitrogen, Carlsbad CA, USA). The optical density at 570 nm was measured using a microplate reader.

### Flow cytometry

EpCAM expression levels were measured by flow cytometry using phycoerythrin-labeled EpCAM antibody with a FACScan flow cytometer (BD Biosciences, San Jose, CA, USA; #347211). EpCAM expression was quantified as mean fluorescence intensity (MFI).

### Luciferase reporter assay

The luciferase reporter constructs pTA-Luc (empty vector control), pTA-AP-1-Luc (AP-1 reporter), and pRL-TK-Luc (transfection control) were obtained form Clontech Laboratories (Mountain View, CA, USA). A 400 ng sample of pTA-Luc or pTA-AP-1-Luc and 20 ng of pRL-TK-Luc were transiently transfected into cell lines, plated in triplicate using either Lipofectamine-LTX (Invitrogen Carlsbad CA, USA) or FuGENE-HD (Roche, Indianapolis, IN, USA). The next day, cells were incubated with serum-free media. After 24 hours, cells were stimulated with phorbol-myristate acetate (PMA), and/or epidermal growth factor (EGF) for an additional 16 hours as indicated. Reporter activity was determined using the Dual-Luciferase kit (Promega, Madison, WI, USA). Reporter activity was measured as luminescence using a luminometer and quantified as relative light units (RLU).

### cDNA synthesis and real-time RT-PCR analysis

RNA was purified from cell lines using RNAeasy (Qiagen, Valencia, CA, USA). Three micrograms of RNA was reverse transcribed using a copy DNA (cDNA) synthesis kit (Ambion, Austin, TX, USA). Quantitative mRNA expression was measured using SYBR green chemistry and an ABI Prism 7700 Sequence Detector (Applied Biosystems, Foster City CA, USA). Primer sequences of genes are available upon request. Each reaction was performed in triplicate, and the data is representative of two independent RNA preparations.

### Multiplex phosphoprotein assay

For the multiplex phosphoprotein assay, CA1a cells were starved for 12 hours in serum-free media and treated with and without 100 ng/mL EGF for 15 minutes. Protein lysates were prepared using a cell lysis kit (Bio-Rad, Hercules, CA, USA). Protein concentration was measured using a BCA protein assay (Pierce, Rockford, IL, USA). The presence of phosphorylated Akt (Ser473), c-Jun (Ser63/73), and JNK (Thr183/Tyr185) were detected by multiplex phosphoprotein assay (Bio-Rad, Hercules, CA, USA) according to the manufacturer's protocol. Data are expressed as mean ± standard error of the mean of triplicate values from separate experiments.

### Immunoblots

For phosphoprotein immunoblots, cells were cultured overnight in serum-free media and stimulated with 25 μg/mL anisomycin for 30 minutes, washed with ice-cold PBS and lysed in cell lysis buffer (Cell Signaling Technology, Danvers, MA, USA). The protein concentration was measured using the BCA protein assay (Pierce, Rockford, IL, USA). A 30 to 50 μg sample of protein was subjected to SDS-PAGE (NuPAGE, Invitrogen, Carlsbad, CA, USA), and transferred by electrophoresis to a polyvinylidene fluoride (PVDF) membrane. Antibodies were obtained from Santa Cruz Biotechnology (Santa Cruz, CA, USA) (EpCAM, #sc-25308; JNK, #sc-571, #sc-474; c-Jun, #sc-1694; HA, #sc-7392; actin, #sc-1615), Sigma-Aldrich (Saint Louis, MO, USA) (FLAG, #F3165), and Cell Signaling Technology (Danvers, MA, USA) (phospho-JNK, #9255; phospho-c-Jun ser73, #9164; SOD1, #2770). Signal detection was performed using the ECL chemiluminescent immunodetection system (Applied Biosystems, Foster City, CA, USA). Immunoblot band density was analyzed using a Bio-Rad GS-800 calibrated densitometer (Bio-Rad, Hercules, CA, USA). For JNK immunoprecipitation, confluent cells were serum starved overnight and immunoprecipitation was carried out as described [33]. To quantify band density, immunoblots were developed on film and then scanned and analyzed using ImageJ software (National Institutes of Health, USA; http://rsbweb.nih.gov/ij/). Plots of each lane were generated, and the area under the peak corresponding to control samples (SCR) was determined, and arbitrarily set at 1.0. The area under the peak corresponding to experimental samples (sh2) was then determined, and expressed relative to the corresponding control.

### Statistical analysis

The data are given as the mean values ± standard deviation. Statistical significance was evaluated by the Student's *t *test. *P *values less than 0.05 were considered to be statistically significant. Significant results are indicated in the appropriate figures with an asterisk.

## Results

### EpCAM expression is associated with breast cancer invasion *in vitro *and *in vivo *

**Figure 1:**
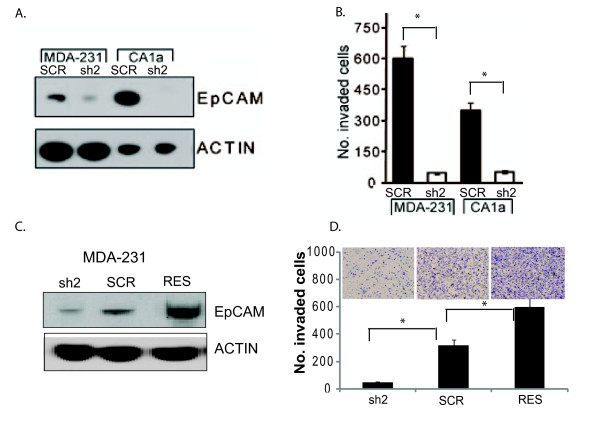
**EpCAM expression is associated with breast cancer invasion *in vitro***. Epithelial cell adhesion molecule (EpCAM) expression was specifically ablated in the breast cancer cell lines MDA-231 and CA1a by RNA interference and rescued, as indicated, by transient transfection with an EpCAM cDNA resistant to RNA interference. **(a) **Specific ablation of EpCAM by shRNA results in a significant decrease in EpCAM protein expression as measured by immunoblot. For each cell line, sh2 designates transduction with a lentiviral vector targeting the 3' untranslated region of EpCAM, while scrambled control shRNA construct (SCR) designates transduction with a lentiviral vector expressing a scrambled control shRNA sequence. (**b) **Invasion was measured using a matrigel transwell invasion assay. After 24 to 72 hours, cells invading through the matrigel membranes were fixed using 70% ethanol, stained with 0.1% crystal violet and photographed in four fields to cover the entire area. Error bars represent the standard error of three experiments performed in triplicate. (**c and d) **Rescue of EpCAM expression in MDA-231 cells by cDNA expression (RES), rescues protein expression (immunoblot) and invasion (matrigel transwell invasion assay).

We have previously reported that specific ablation of EpCAM decreases breast cancer invasion *in vitro *[4,7,32]. Many of these studies were performed with siRNA, and the short duration of RNA interference associated with these reagents precluded *in vivo *and/or mechanistic experiments. To confirm the impact of EpCAM on breast cancer invasion *in vivo*, and to establish a functional rescue/gain-of-function model system for mechanistic studies, we generated stable breast cancer cell lines with specific ablation of EpCAM expression using EpCAM-specific lentiviral shRNA constructs. Stable transduction of breast cancer cell lines with EpCAM-specific lentiviral shRNA constructs results in more than 90% reduction in EpCAM protein expression as measured by immunoblot (Figure 1a). Stable specific ablation of EpCAM in both MDA-231 and CA1a breast cancer cell lines is also associated with a more than 80% decrease in invasion in matrigel transwell invasion assays (Figure 1b).

To control for off-target effects of RNA interference, and establish a functional rescue model system for mechanistic studies, we specifically ablated EpCAM expression using specific shRNA constructs and then rescued EpCAM expression using EpCAM cDNA constructs resistant to RNA interference as we previously described [32] (Figures 1c and 1d). In these experiments, EpCAM rescue in MDA-231 and CA1a breast cancer cells results in EpCAM overexpression as measured by immunoblot, and increased invasion, establishing a gain-of-function model system.

**Figure 2:**
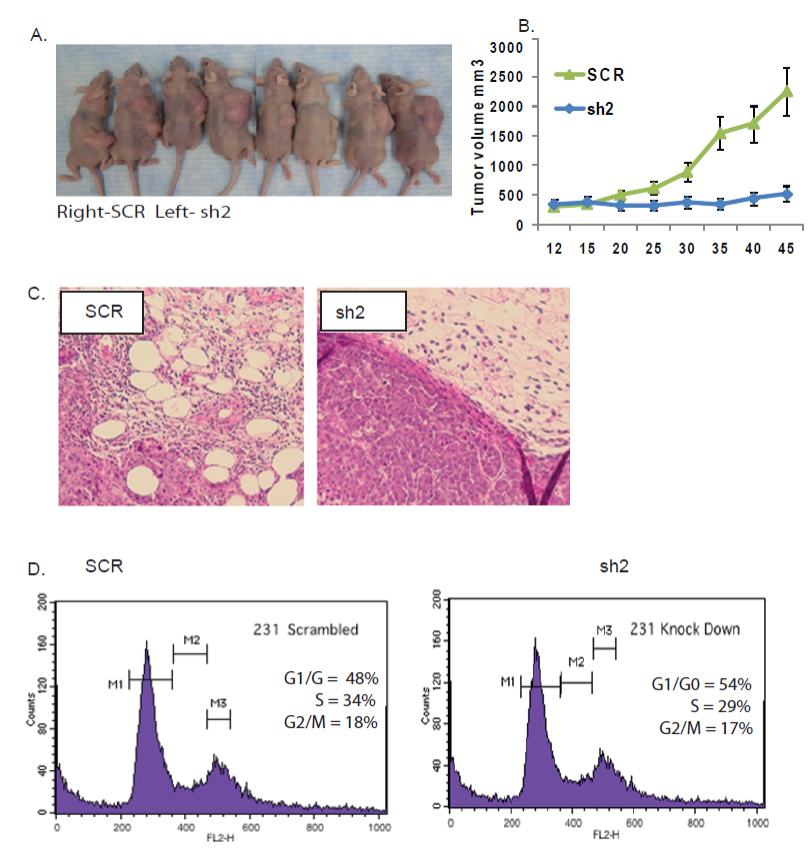
**EpCAM expression is associated with breast cancer invasion *in vivo *in a breast cancer xenograft model**. **(a and b) **MDA-231 breast cancer cells were stably transduced with experimental (sh2) or control (scrambled control shRNA construct (SCR)) shRNA lentiviral constructs. One × 10^7 ^MDA-231 cells were resuspended in matrigel (1:1 ratio by volume) and injected into nude mice (*n *= 10 per group). The right flank was injected with MDA-231(SCR) cells and the left flank was injected with MDA-231(sh2) cells. Tumor size was measured by electronic calipers every thre to five days. Photos at the time of sacrifice demonstrate tumor growth in the right flank but not in the left flank. **(c) **H&E evaluation of the tumor site demonstrates that MDA-231(SCR) cells were invading into the surrounding fat, while MDA-231(sh2) tumors were not invasive. **(d) **Specific ablation of EpCAM did not impact cell cycle/proliferation of MDA-231 cells when analyzed prior to tumor challenge. The percentage of cells in each stage of the cell cycle is indicated.

We used the lentiviral vectors validated above to determine the impact of EpCAM expression on breast cancer invasion in vivo, taking advantage of a commonly used breast cancer xenograft model [34-36]. Specific ablation of EpCAM expression in MDA-231 breast cancer cells significantly decreases tumor growth following tumor challenge in nude mice (Figures 2a and 2b). Histological examination of the tumors confirms that specific ablation of EpCAM abrogates the ability of tumors to invade into surrounding tissues (Figure 2c). Of note, we and others have observed that depending on the experimental conditions, EpCAM expression can impact proliferation [3-5]. Significant changes in proliferation could impact interpretation of the *in vivo *tumor challenge studies. However, only minimal changes in cell cycle (Figure 2d) and proliferation (data not shown) were observed following specific ablation of EpCAM expression in MDA-231 breast cancer cells under these experimental conditions.

**Figure 3:**
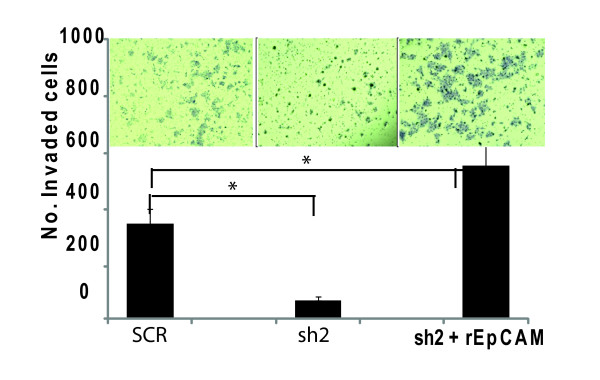
**Recombinant extracellular EpCAM rescues breast cancer invasion *in vitro***. To evaluate the impact of epithelial cell adhesion molecule (EpCAM) signaling on breast cancer invasion, rEpCAM (200 ng/mL) or vehicle was mixed with MDA-231(sh2) cells and plated on top matrigel chamber. After 48 hours, the cells were harvested and invasion was evaluated. The results are representative of two independent experiments.

Recently, Gires et al. demonstrated that EpCAM is cleaved by regulated intramembrane proteolysis, resulting in release of the extracellular domain as a soluble ligand, and translocation of the intracellular domain (EpICD) into the nucleus [5,37]. In these studies, the extracellular domain of EpCAM is also able to modulate EpCAM signaling. We used recombinant soluble extracellular EpCAM (rEpCAM) to rescue EpCAM signaling following specific ablation of EpCAM. Addition of rEpCAM to MDA-231 breast cancer cells is able to rescue invasion following specific ablation of EpCAM (Figure 3). Taken together, these findings confirm the hypothesis that EpCAM expression modulates breast cancer invasion, with increased EpCAM expression and/or signaling associated with increased breast cancer invasion.

### EpCAM expression is associated with increased AP-1 transcription factor activity in breast cancer cells

**Figure 4:**
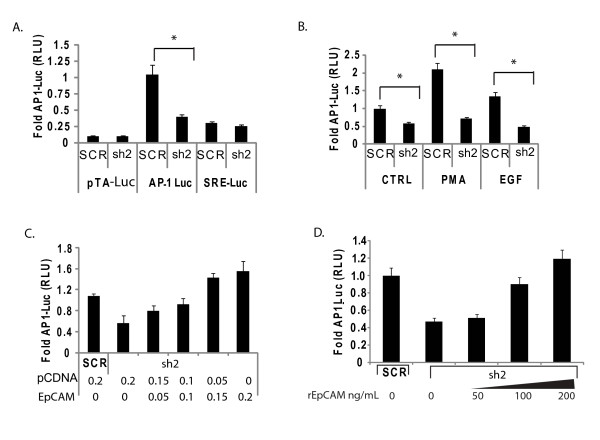
**EpCAM expression is associated with AP-1 transcription factor activity**. Epithelial cell adhesion molecule (EpCAM) expression was specifically ablated in breast cancer cells using lentiviral constructs, and then rescued by cDNA or recombinant EpCAM. **(a) **A luciferase reporter pathway profiling system was used to measure the impact of EpCAM expression on the activity of specific transcription factors. Results from the pTA (control), activator protein 1 (AP-1) and SRE reporters are shown. AP-1 transcription factor activity was significantly decreased following specific ablation of EpCAM in MDA-231(sh2) cells. Other transcription factors were evaluated but specific ablation of EpCAM had minimal effect (data not shown). Stably transduced MDA-231 cells (scrambled control shRNA construct (SCR) and sh2) were plated for 12 hours, then transiently transfected with the reporters indicated. Luciferase activity was measured 48 hours later. (**b) **Following this same protocol, the AP-1 reporter was transfected into stably transduced MDA-231 cells (SCR and sh2). Twelve hours after transfection, AP-1 transcription factor activity was induced with phorbol-myristate acetate (PMA; 50 ng/mL) or epidermal growth factor (EGF; 10 ng/mL). 12 hours after induction, reporter activity was measured. (**c) **AP-1 transcription factor activity is rescued in a dose-dependent fashion by transfection with increasing concentrations of EpCAM cDNA. MDA-231(sh2) cells were transiently transfected with 200 ng of pcDNA3, or 50 to 200 ng of pCDNA-EpCAM as indicated. The total amount of DNA was constant at 200 ng, as indicated. (**d) **AP-1 transcription factor activity is rescued in a dose-dependent fashion by recombinant EpCAM. Twelve hours after transient transfection with the AP-1 reporter, the indicated doses of rEpCAM were added to MDA-231(sh2) cells. Fourteen hours after addition of rEpCAM, AP-1 reporter activity was measured. The results are representative of at least two independent experiments.

To address the potential mechanism(s) underlying the impact of EpCAM on breast cancer invasion, we used a luciferase reporter pathway profiling system to measure the activity of specific transcription factors following specific ablation of EpCAM expression. In this system, individual vectors in the profiling system contain the transcriptional response elements of the transcription factors of interest upstream of a luciferase reporter. These reporters are then individually transfected with a control TK-*Renilla *construct into cells stably transduced with control or EpCAM-specific shRNA vectors. In these studies, we focused on the AP-1 transcription factor, because AP-1 has been shown to be a critical regulator of a complex program of gene expression contributing to the invasive phenotype [38]. Using this strategy, we found that specific ablation of EpCAM is associated with a two-fold or greater reduction in AP-1 transcription factor activity in MDA-231 breast cancer cells at baseline (Figure 4a), or in the presence of EGF, or PMA, which are known to induce AP-1 activity [39] (Figure 4b). Rescue of EpCAM expression using EpCAM cDNA resistant to RNA interference, or rescue of EpCAM signaling using recombinant soluble EpCAM resulted in a dose-dependent rescue of AP-1 transcription factor activity, confirming the specificity and significance of these observations (Figures 4c and 4d).

### EpCAM expression is associated with increased phosphorylation of the AP-1 protein subunit c-Jun

**Figure 5:**
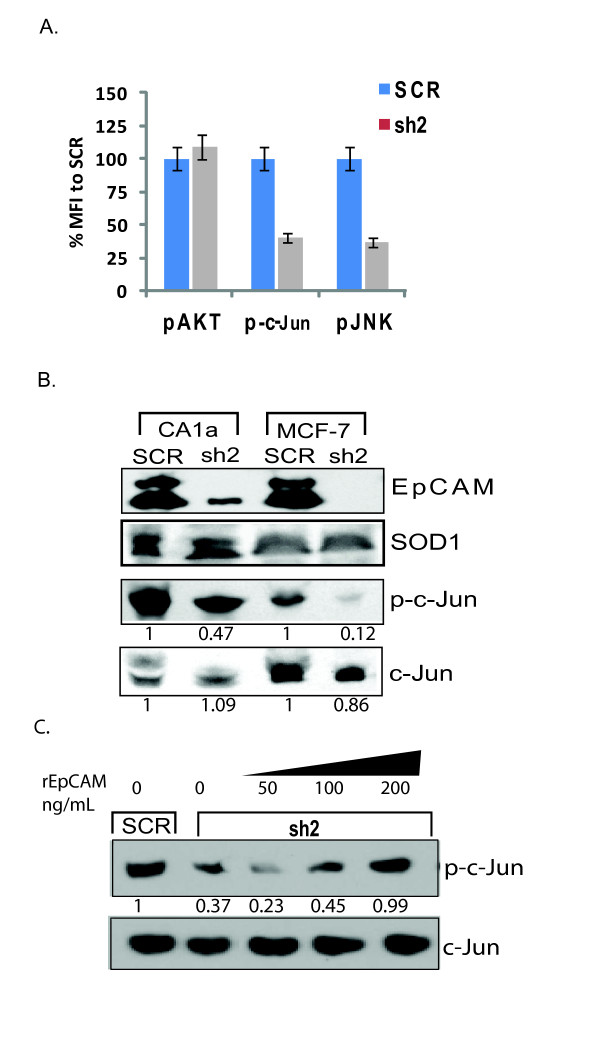
**EpCAM expression is associated with phosphorylation of the AP-1 transcription factor protein subunit c-Jun**. **(a) **Specific ablation of epithelial cell adhesion molecule (EpCAM) is associated with decreased c-Jun phosphorylation as measured by multiplex phosphoprotein assay. Stably transduced CA1a cells (scrambled control shRNA construct (SCR) and sh2) were induced with epidermal growth factor (EGF), and phosphorylation was measured using a multiplex phosphoprotein assay. Relative levels of phosphorylated (p)-AKT, p-c-Jun, and p-JNK were measured by flow cytometry and expressed as mean fluorescence intensity (MFI). Error bars represent standard error. **(b) **Specific ablation of EpCAM is associated with decreased c-Jun phosphorylation as measured by phosphoprotein immunoblot. Stably transduced CA1a and MCF-7 cells (SCR and sh2) were serum-starved for six hours. The cells were treated with 20 μM anisomycin for 15 minutes and 30 μg of the cell lysate was analyzed with antibodies recognizing EpCAM, superoxide dismutase (SOD, loading control), p-c-Jun-63, or total c-Jun. Relative band density was quantified using ImageJ software, with the results indicated below the relevant band. **(c) **Recombinant EpCAM is capable of rescuing c-Jun phosphorylation in CA1a(sh2) cells. CA1a(sh2) cells were serum-starved for six hours and then treated with rEpCAM at concentrations of 50, 100, and 200 ng/mL for two hours. The cells were then stimulated with anisomycin for 10 minutes. A 30 μg sample of each cell lysate was analyzed for p-c-Jun and total c-Jun. Relative band density was quantified using ImageJ software, with the results indicated. The results are representative of two independent experiments.

AP-1 can be activated through at least two possible regulatory mechanisms [40,41]. First, transcription of c-Jun and other AP-1 protein subunits can be rapidly induced, resulting in increased total protein levels. Second, AP-1 protein subunits can be phosphorylated by mitogen-activated protein kinases (MAPKs), including JNK. In preliminary studies using a multiplex phosphoprotein assay, specific ablation of EpCAM decreased c-Jun and JNK1 phosphorylation in breast cancer cells (Figure 5a). To confirm and extend these findings, we specifically ablated EpCAM in breast cancer cells (CA1a, and MCF-7), and then measured the levels of total and phosphorylated c-Jun by protein immunoblot and densitometry. In CA1a and MCF-7 breast cancer cells, specific ablation of EpCAM decreased phosphorylated c-Jun, but had no significant impact on total c-Jun (Figure 5b). A similar decrease in phosphorylated but not total ATF2 was also observed (data not shown). We also evaluated total and phosphorylated c-Jun following rescue of EpCAM signaling by rEpCAM. Increasing doses of rEpCAM rescued phosphorylated c-Jun following specific ablation of EpCAM in CA1a cells (Figure 5c). Taken together, these studies suggest that phosphorylation of existing AP-1 protein subunits contributes to modulation of AP-1 transcription factor activity following specific ablation or rescue of EpCAM expression and/or signaling.

### The JNK signal transduction pathway contributes to EpCAM-dependent AP-1 transcription factor activity

**Figure 6:**
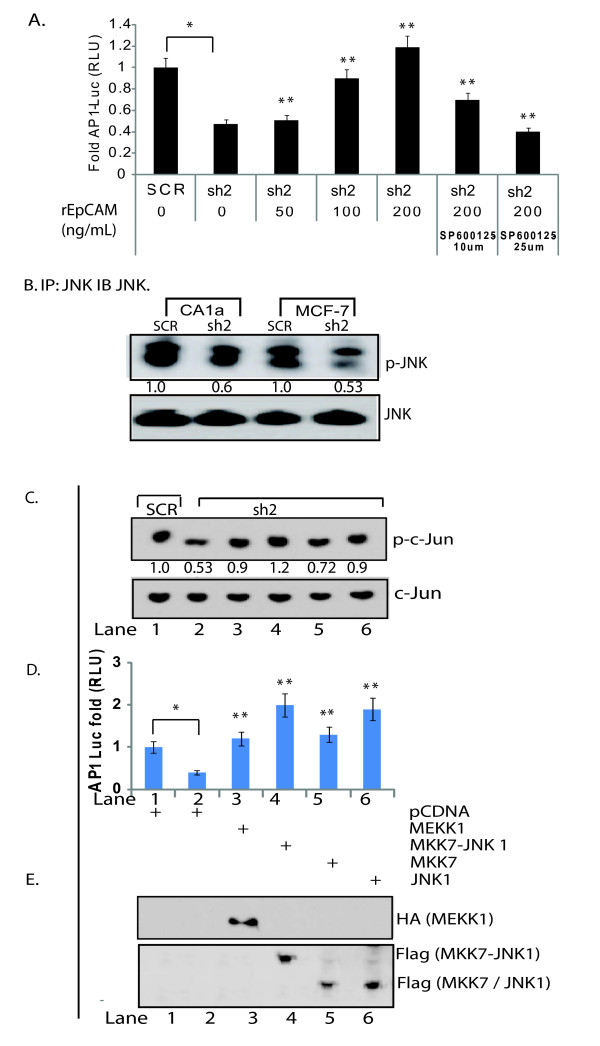
**The MEKK1/MKK7/JNK signal transduction pathway contributes to EpCAM-dependent AP-1 transcription factor activity**. **(a) **Pharmacologic inhibition of c-Jun N-terminal kinase (JNK) abrogates recombinant soluble extracellular epithelial cell adhesion molecule (rEpCAM)-mediated rescue of activator protein 1 (AP-1) transcription factor activity. Stably transduced CA1a (scrambled control shRNA construct (SCR) and sh2) cells were transiently transfected with AP-1 and control luciferase reporters. Six hours after transfection, cells were treated with SP600125 (JNK inhibitor) at 10 μM and 20 μM for one hour followed by addition of rEpCAM. Luciferase reporter activity was measured 16 hours later. **(b) **To directly evaluate JNK phosphorylation, SCR and sh2 CA1a and MCF-7 cells were serum starved for 12 hours, stimulated with 20 μM anisomycin for 10 minutes, and then immunoprecipitated overnight with a JNK antibody. JNK phosphorylation was evaluated by immunoblot using antibodies specific for p-JNK1/2 and total JNK1. Relative band density was quantified using ImageJ software, with the results indicated. **(c) **Constitutively active genetic constructs corresponding to mitogen activated protein (MAP) kinases in the JNK signal transduction pathway rescued c-Jun phosphorylation and AP-1 transcription factor activity following specific ablation of EpCAM in MCF-7 breast cancer cells. Stably transduced SCR or sh2 MCF-7 cells were transiently transfected with plasmids encoding HA-MEKK1, FLAG-MKK7-JNK1, FLAG-MKK7, and FLAG-JNK1. After 16 hours, cells were analyzed by immunoblot for c-Jun phosphorylation. Relative band density was quantified using ImageJ software, with the results indicated. **(d) **Duplicate samples were transiently transfected with control and AP-1 luciferase reporters, and AP-1 transcription factor activity was measured. **(e) **Expression of HA-MEKK1, FLAG-MKK7-JNK1, FLAG-MKK7, and FLAG-JNK1 was confirmed by immunoblot with antibodies specific for the indicated protein tag (HA or FLAG). The results are representative of two independent experiments. For AP-1 reporter assay in Figure a and d, *P *< 0.05 were considered to be statistically significant when compared between SCR, sh2 (*) and sh2, sh2 treated/transfected cells (**).

AP-1 is a major target of the JNK signal transduction pathway, and activated JNK can bind and phosphorylate c-Jun. To directly evaluate the impact of EpCAM expression on JNK phosphorylation, we manipulated EpCAM expression and then measured JNK phosphorylation by immunoblot and densitometry. Specific ablation of EpCAM results in a significant decrease in JNK phosphorylation in CA1a and MCF-7 breast cancer cells (Figure 6a). To evaluate the contribution of the JNK signal transduction pathway in more detail, we used pharmacologic inhibitors of the JNK signal transduction pathway. Specific ablation of EpCAM is associated with a decrease in AP-1 transcription factor activity, and this decrease can be rescued in a dose-dependent fashion with rEpCAM (Figure 6a). Addition of SP600125, an anthrapyrazolone inhibitor of JNK [42], abrogates the increase in AP-1 transcription factor activity induced by rEpCAM, suggesting a role for the JNK signal transduction pathway. To confirm and extend these findings we used constitutively active genetic constructs corresponding to MAP kinases in the JNK signal transduction pathway (MEKK1-MKK7-JNK). Constitutively active JNK MAPK constructs rescued c-Jun phosphorylation (Figure 6c) and AP-1 transcription factor activity (Figure 6d) following specific ablation of EpCAM in MCF-7 breast cancer cells. Taken together these studies provide evidence that EpCAM expression can modulate the JNK signal transduction pathway, and AP-1 transcription factor activity.

### AP-1 contributes to EpCAM-mediated breast cancer invasion

**Figure 7:**
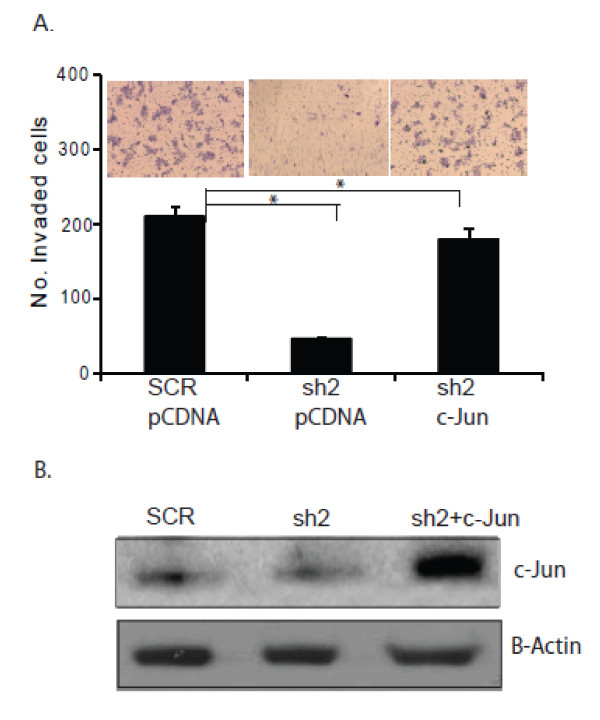
**Forced overexpression of c-Jun rescues breast cancer invasion *in vitro *following specific ablation of EpCAM**. **(a) **Stably transduced CA1a cells (sh2), were transiently transfected with pcDNA or c-Jun constructs. After 24 hours, invasion was assessed using a matrigel transwell invasion assay. **(b) **Transfected cells were analyzed for c-Jun mRNA expression by RT-PCR. Actin mRNA expression served as a control for RT-PCR conditions. The results are representative of two independent experiments. EpCAM, epithelial cell adhesion molecule.

AP-1 is considered to be a central transcription factor in the regulation of cancer invasion [38]. To directly assess the functional contribution of AP-1 in EpCAM-mediated breast cancer invasion, we specifically ablated EpCAM expression in CA1a breast cancer cells, and then rescued AP-1 transcription factor activity using a constitutively active c-Jun genetic construct [43]. c-Jun expression is able to efficiently rescue AP-1 transcription factor activity (data not shown) and invasion (Figure 7a), suggesting that the AP-1 protein subunit c-Jun is a key downstream mediator of EpCAM biology, contributing to EpCAM-dependent breast cancer invasion.

### EpCAM expression modulates the expression of AP-1 target genes known to be involved in cancer invasion

**Figure 8:**
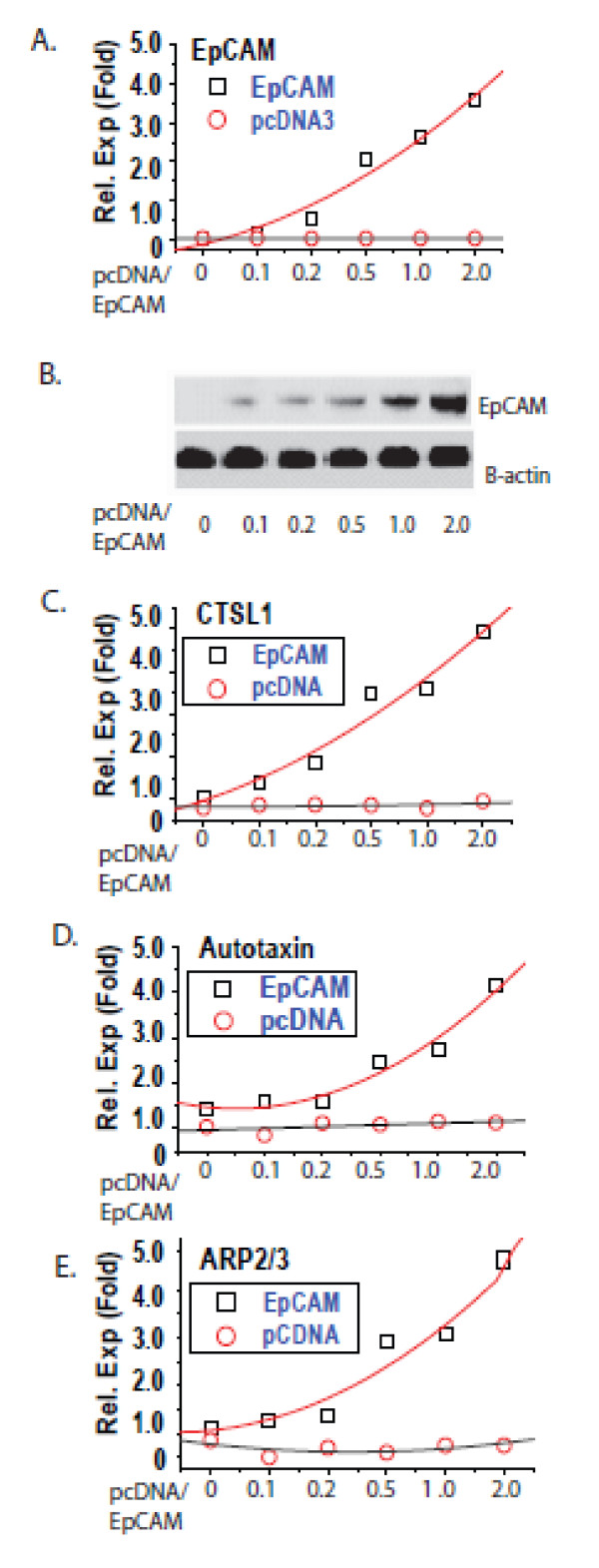
**EpCAM expression modulates expression of AP-1 target genes known to be involved in cancer invasion**. MDA-231 cells were transiently transfected with control and epithelial cell adhesion molecule (EpCAM) cDNA expression constructs and allowed to stabilize for 24 hours. The cells were then serum-starved for 16 hours, and then stimulated with 10% FBS DMEM growth media for two hours. RNA was extracted using RNeasy (Qiagen). Two micrograms of RNA was reverse transcribed and 40 ng equivalent cDNA was used for each assay in triplicate. Activator protein 1 (AP-1) target genes CTSL1, Autotaxin, and ARP2/3 were analyzed using real-time PCR. Relative expression was quantified using the [delta] [delta] Ct method with 18S rRNA as a control. The results are representative of at least two independent experiments.

To confirm the impact of EpCAM on AP-1 target gene expression, we specifically ablated EpCAM and then analyzed more than 120 AP-1 target genes known to be involved in cancer invasion using quantitative RT-PCR [38]. We identified more than 30 genes that were impacted at the mRNA level by specific ablation of EpCAM. To confirm these results, EpCAM expression was specifically ablated in CA1a breast cancer cells, and then rescued with increasing doses of EpCAM cDNA. Increasing EpCAM expression is associated with a dose-dependent increase in the expression of the AP-1 target genes CTSL1, Autotaxin and ARP2/3 (Figure 8). These studies provide additional support for the hypothesis that EpCAM expression modulates AP-1 transcription factor activity.

## Discussion

EpCAM is a cell-surface glycoprotein that is overexpressed on the majority of epithelial cancers, including breast cancer. However, the functional role of EpCAM in cancer invasion remains controversial. In this study we focused on the role of EpCAM in breast cancer invasion. We confirm that EpCAM expression is associated with increased breast cancer invasion *in vitro *and *in vivo*, and demonstrate for the first time that EpCAM expression is capable of modulating the JNK/AP-1 signal transduction pathway. In functional rescue experiments, we demonstrate that forced overexpression of c-Jun is capable of rescuing breast cancer invasion following specific ablation of EpCAM, confirming that AP-1 is a key downstream mediator of EpCAM-dependent breast cancer invasion.

Recently, Munz et al. demonstrated that *de novo *induction of EpCAM expression is associated with the rapid upregulation of c-myc expression in epithelial cells, strongly suggesting that EpCAM can function as a signaling molecule [3]. In their studies the most dramatic phenotype of forced EpCAM expression was on cell cycle and proliferation, consistent with the known role of c-myc in regulating genes involved in control of the cell cycle. However, we and others have found that the most dramatic phenotype associated with manipulation of EpCAM expression in human cancers is altered invasion [4,7,29,30]. This suggests that other signaling pathways may also be modulated by EpCAM expression/signaling. Our finding that EpCAM differentially regulates the JNK/AP-1 signal transduction pathway is particularly relevant in this context. The AP-1 transcription factor is a heterodimeric protein composed of proteins belonging to the c-Jun, c-Fos, ATF, and JDP families [39]. AP-1 is activated in response to a variety of stimuli, including cytokines, growth factors, stress, and infection, and is considered to be a central transcription factor in the regulation of cell invasion [38]. Of particular note, AP-1 appears to be overexpressed in breast cancer [44,45], and is currently being evaluated as a target for molecular therapy in this disease [46-48]. Recent studies have focused specifically on the role of c-Jun in breast cancer. In primary breast cancers, activated c-Jun is present at the invasive front, and is associated with proliferation and angiogenesis [45]. In addition, recent studies confirm the role of c-Jun in breast cancer invasion *in vitro *[49], and in spontaneous tumors derived from genetically engineered mice [50]. Despite the central role of AP-1 as a regulator of invasion, our studies do not exclude the possibility that other signaling pathways are modulated by EpCAM, and may also contribute to the regulation of invasion in breast cancer. Recently, Gostner et al. demonstrated that specific ablation of EpCAM in MDA-231 cells may modulate the Wnt signaling pathway [51].

Maetzel et al. recently demonstrated that EpCAM is cleaved by regulated intramembrane proteolysis in a hypopharyngeal cancer cell line, resulting in the release of the extracellular portion of the molecule, Ep^EX^, and the intracellular domain, Ep^IC ^[5]. In their studies, Ep^IC ^localizes to the nucleus, and interacts with FHL2 and β-catenin to promote Wnt signaling. Our studies confirm the importance of the extracellular domain of EpCAM in EPCAM signaling; addition of recombinant extracellular EpCAM rescues breast cancer invasion, AP-1 transcription factor activity, and c-Jun phosphorylation in a dose-dependent fashion following specific ablation of EpCAM. However, we have been unable to rescue invasion by forced overexpression of Ep^IC ^(data not shown). FHL2 is known to be an inducible co-activator of AP-1 [52], and one hypothesis is EpCAM expression/signaling results in Ep^IC ^translocation to the nucleus and subsequent interaction with FHL2 and AP-1 proteins to increase AP-1 signaling. Alternatively, EpCAM expression/signaling may modulate the JNK signal transduction pathway directly or indirectly, leading to c-jun phosphorylation and AP-1 activation. In support of this latter hypothesis, cell adhesion molecules such as ICAM-1 are known to modulate the JNK/AP-1 signaling pathway. Although our data does suggest that the JNK/AP-1 signaling pathway is modulated, these hypotheses are not mutually exclusive, and additional studies are ongoing to determine how EpCAM signaling impacts AP-1 transcription factor activity.

Finally, EpCAM represents an attractive target for molecular therapy in epithelial carcinomas. EpCAM is overexpressed in the majority of epithelial carcinomas, and there are currently a number of different molecular therapies under development [1]. The results of the studies presented here have important implications for the design and application of these molecular therapies. The most important implication is that molecular therapies under investigation should be evaluated for their ability to influence EpCAM-mediated signaling pathways, as recent studies suggest that monoclonal antibodies targeting EpCAM may alter EpCAM signaling [53]. Molecular therapies capable of interfering with EpCAM-mediated signaling may find particular success in the treatment of breast cancer, where EpCAM expression is associated with increased invasion and poor prognosis.

## Conclusions

EpCAM expression is associated with increased breast cancer invasion. In mechanistic studies, we demonstrate that EpCAM expression modulates the JNK signal transduction pathway, and AP-1 transcription factor activity in breast cancer cells. Functional studies confirm that AP-1 is a key downstream mediator of EpCAM biology, contributing to EpCAM-dependent breast cancer invasion. Further study of EpCAM-mediated signaling pathways is necessary to facilitate the rational design and application of molecular therapies targeting EpCAM.

## Abbreviations

AP-1: activator protein 1; BSA: bovine serum albumin; cDNA: copy DNA; EGF: epidermal growth factor; EpCAM: epithelial cell adhesion molecule; Ep^EX^: EpCAM extracellular domain; Ep^IC^: EpCAM intracellular domain; H&E: hematoxylin and eosin; JNK: c-Jun N-terminal kinase; MAPK: mitogen activated protein kinase; MFI: mean fluorescent intensity; PBS: phosphate-buffered saline; PMA: phorbol-myristate acetate; rEpCAM: recombinant soluble extracellular EpCAM; RLU: relative light unit; RT-PCR: reverse transcription polymerase chain reaction; SCR: scrambled control shRNA construct; shRNA: short hairpin RNA; sh2 = shRNA construct targeting the untranslated region of EpCAM mRNA.

## Competing interests

The authors declare that they have no competing interests.

## Authors' contributions

NVS contributed to the conception of the study, performed the majority of the molecular biology studies, and wrote the first draft of the manuscript. JDM performed some of the phosphorylation studies and assisted in other molecular biology experiments. MWW participated in the rEpCAM signaling experiments and assisted in cloning many of the constructs used in the study. TPF performed the invasion assays, contributed to study conception, data interpretation and critical manuscript review. WEG contributed to study conception, data interpretation, troubleshooting, and edited the manuscript. All authors read and approved the final manuscript.

## Authors' information

JDM is currently a post-doctoral fellow at the Duke Institute for Genome Sciences & Policy (IGSP). MWW is currently a medical student at Saint Louis University.
